# Combination of Medical and Surgical Treatment in Scleromyxedema

**DOI:** 10.7759/cureus.32729

**Published:** 2022-12-20

**Authors:** Maria Caridad Duran-Lemarie, Luis Enrique Cano-Aguilar, Cristina Berumen-Glinz, Zonia Quijada-Ucelo, Heidi Hernandez-Ramirez, Mariana De Anda-Juárez, Elisa Vega-Memije

**Affiliations:** 1 Dermatology, General Hospital “Dr. Manuel Gea González”, Mexico City, MEX; 2 Dermatopathology, General Hospital “Dr. Manuel Gea González”, Mexico City, MEX

**Keywords:** mucin, fibromucinous dermatosis, skin surgery, lichen myxedematosus, scleromyxedema

## Abstract

Scleromyxedema is an uncommon and progressive fibromucinous disorder characterized by disseminated papular eruption with histological features of dermal mucin deposition. The skin changes associated with this disease are highly visible and they tend to affect the patient’s quality of life. We report a case of a 50-year-old male patient that presented a 3-year-old history of disseminated asymptomatic firm papules-associated systemic symptoms. Medical treatment with oral corticosteroid and thalidomide was indicated and surgical treatment on residual facial folds was performed, with an excellent outcome.

## Introduction

Scleromyxedema (SM) is an uncommon chronic and progressive fibromucinous skin disorder of unknown etiology that was first described by Arndt and Gottron in 1954 [1,2]. SM is characterized by a generalized papular or nodular eruption with indurated perilesional skin. The histopathologic features of SM are excessive mucin deposition and fibroblast proliferation [3]. The quality of life may be affected because facial folds may be distorted.

We report a case of SM causing an important facial disfigurement that was treated with systemic therapy and skin excision with an excellent outcome.

## Case presentation

**Figure 1:**
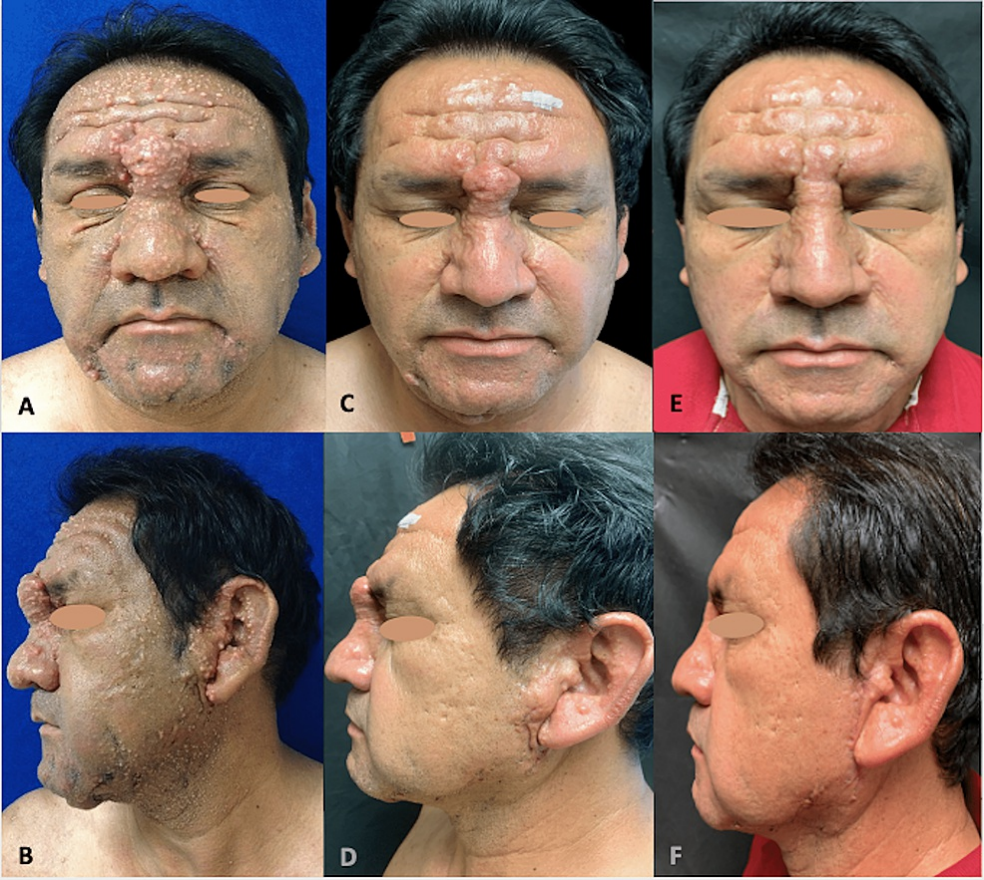
At presentation (A) anterior view, (B) lateral view. One-year follow-up with medical treatment: (C) anterior view; (D) lateral view. Three-month follow-up after surgical treatment: (E) anterior view; (F) lateral view.

**Figure 2:**
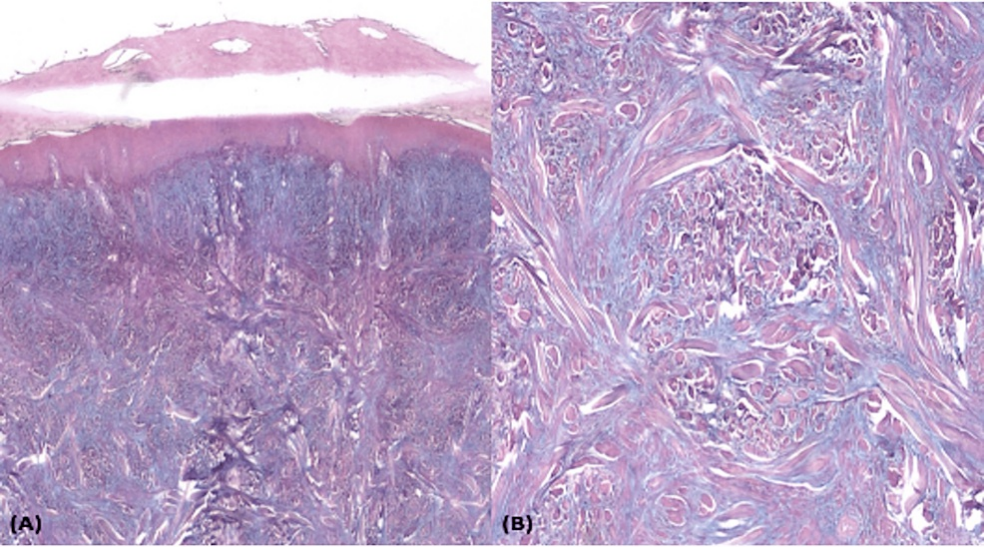
Pathology images Superficial and deep fibrosing dermatitis with interstitial mucin. (A) Stain type: Alcian blue, magnification 4x. (B) Stain type: Alcian blue, magnification 10x.

A 50-year-old male patient, with no significant medical history, presented with a 3-year-old history of asymptomatic, firm papules disseminated to all body parts, associated with arthralgia, decreased movement of the joints, muscle weakness, and visual impairment. Physical examination revealed multiple asymptomatic and symmetrical, translucent, skin-red colored, dome-shaped, firm papules of 2-3 mm. Perilesional skin was shinny and indurated. On the face, some of those papules coalesced in elevated plaques with deep glabellar lines that reassembled leonine facies. A central depression surrounded by a raised edge on proximal interphalangeal hand joints was also seen (known as the “doughnut sign”). On the back, the papules were distributed in a linear pattern with deeper furrows and redundant skin folds (known as the “Shar-Pei sign”) (Figures 1A-1D). A skin biopsy showed tapered cells in fascicle arrangements, giving a swirling appearance in the dermis. These cells had elongated nuclei, blunt tips, fine chromaffin patterns without nuclear atypia, some of them were multinucleated and interspersed with thickened and compacted collagen fibers (Figures 2A-2B). The inflammatory infiltrate was composed of lymphocytes and plasma cells. This superficial and deep fibrosing dermatitis with interstitial mucin was compatible with SM. Prednisone at 1 mg/kg per day (80 mg) and 100 mg/day of thalidomide were started. Our patient presented clinical improvement and only the glucocorticoid therapy was suspended after careful tapering, but redundant skin folds on his face persisted (Figures 1C-1D). Surgical excision was performed on the preauricular and glabella skin. The patient remained with thalidomide treatment and without medical complications after 3 months of surgery. He still continues with follow-up visits and thalidomide treatment to monitoring clinical response (Figures 1E-1F).

## Discussion

SM is an uncommon and progressive cutaneous mucinosis with significant morbidity associated with systemic symptoms [1]. It usually affects adults between 30 and 80 years old with no sex or race predominance [4]. The pathogenesis of the disease remains unknown [5]. The main hypothesis is that IL-1, TNF-alpha, and transforming growth factor (TGF)-beta cytokines from the bone marrow stimulate glycosaminoglycan synthesis and fibroblast proliferation in the dermis [6,7].

SM is described as a widespread eruption of 2-3 mm firm, flesh-colored, spaced, dome-shaped papules arranged in a linear array with shiny and indurated surrounding skin [6,8]. Deep longitudinal furrows can be found in the glabella, producing the distinctive leonine face and microstomia [6]. These deep furrows are also seen on the trunk and limbs known as the Shar-Pei sign [6]. As the disease evolves, infiltrated plaques may stiffen the skin producing sclerodactyly and decreases the movement of the mouth and joints [6].

Extracutaneous manifestations occur in 63%-77% of patients, resulting from mucin deposition in internal organs [9]. The most common extracutaneous manifestations are neurologic, rheumatologic, and cardiac abnormalities [4].

SM is characterized by the histopathological triad of mucin deposition, fibrosis, and irregularly arranged fibroblast proliferation [6].

There is no accepted consensus on its treatment [9]. All the available data are limited to case reports and relapses occur frequently upon the interruption of medication [6,7]. There are available data on medical treatment, but only a few reports of the surgical management of patients with SM were found [10]. An aggressive surgical approach with excision and dermabrasion had been described [11,12]. In follow-up visits, the patient presented an excellent outcome without recurrence.

## Conclusions

SM is an uncommon chronic and progressive primary mucinosis with an unpredictable course. It is important to consider the combination of medical and surgical treatment due to facial disfigurement and the patient’s quality of life. Here, we present a case of SM with an improvement of systemic symptoms and surgical resolution of facial skin folds. There are still no definitive guidelines on the best treatment option.
